# Molecular Diagnosis of Two Major Implantation Mycoses: Chromoblastomycosis and Sporotrichosis

**DOI:** 10.3390/jof8040382

**Published:** 2022-04-09

**Authors:** Danièle Maubon, Cécile Garnaud, Lala Soavina Ramarozatovo, Rapelanoro Rabenja Fahafahantsoa, Muriel Cornet, Tahinamandranto Rasamoelina

**Affiliations:** 1 Translational Innovation in Medicine and Complexity, Centre National de la Recherche Scientifique, Université Grenoble Alpes, Domaine de la Merci, Centre Hospitalier Universitaire Grenoble Alpes, Service de Parasitologie-Mycologie, Bd de la Chantourne, CEDEX, 38706 La Tronche, France; dmaubon@chu-grenoble.fr (D.M.); cgarnaud@chu-grenoble.fr (C.G.); 2 Université Hospital Joseph Raseta Befelatanana, Antananarivo 101, Madagascar; lsramarozatovo@gmail.com (L.S.R.); frapelanoro@gmail.com (R.R.F.); 3 Centre d’Infectiologie Charles Mérieux, Université d’Antananarivo, Antananarivo 101, Madagascar; ninamandranto@yahoo.fr

**Keywords:** chromoblastomycosis, sporotrichosis, molecular, sequencing, PCR, MALDI-TOF MS, isothermal PCR

## Abstract

Chromoblastomycosis and sporotrichosis are the two main implantation mycoses that are now recognized as fungal neglected tropical diseases (NTDs). Their laboratory diagnosis mainly relies on direct microscopy, histopathology, and identification of the fungus by culture. However, to be appropriately used, these techniques require mycological expertise that is not widely available and may be absent in peripheral health care facilities in endemic areas. In addition, they lack sensitivity and specificity, and the culture for isolation and identification can have a long time-to-results period. Molecular methods, including matrix-assisted laser desorption/ionization time-of-flight mass spectrometry (MALDI-TOF MS), have been developed in well-equipped reference laboratories. They greatly improve the rapidity and accuracy of diagnosis; in particular, for species identification. Recently, PCR and sequencing have paved the way for more user-friendly point-of-care tests, such as those based on LAMP or RCA technologies, which can be used in basic healthcare settings and even in field consultations.

## 1. Introduction

Chromoblastomycosis (CBM) and sporotrichosis (SPT) are implantation mycoses that are now recognized as fungal NTDs. CBM is a member of the phaeohyphomycosis group, together with black-grain mycetoma. CBM is linked to poverty and a lack of protective clothing or shoes for persons working in rural areas in which spiny plants are common. It predominates in tropical and subtropical regions, and most reported cases are from Madagascar, Africa, Latin America (Brazil, Mexico, and Venezuela), the Caribbean (Dominican Republic and Cuba), Asia (India, Japan, and southern China), and Australia [1,2,3,4]. Globally, up to 7740 cases have been reported, mainly in South America (n = 2619), Africa (n = 1875), and Asia (n = 1390) [3]. CBM has a number of clinical and diagnostic features that are pathognomonic and distinguish it from the other phaeohyphomycosis. Indeed, CBM is a chronic fungal infection of the subcutaneous tissues characterized by warty hyperkeratosic plaques, verrucous lesions, and pink pimples with a typical ‘cauliflower’ appearance. The presence of fumagoïd cells on microscopic examination of squamous samples provides rapid and sufficient evidence of the presence of a dematiaceous agent responsible for CBM and may lead to targeted therapy. However, identification of the species, or at least its genus level, is recommended, in order to guide the choice of antifungal molecule [1,5]. The main causative agents of CBM are melanized filamentous fungi of the genus *Fonsecaea* spp., or *Cladophialophora* spp. Rare cases caused by *Rhinocladiella* spp., *Phialophora* spp., or the ‘black yeasts’ *Exophiala* spp. have been reported [1,6,7].

Sporotrichosis (SPT) is a chronic fungal infection, first described by Schenk in 1898, caused by dimorphic fungi of the genus *Sporothrix*. These species show two different morphological appearances, depending on the temperature (i.e., a yeast form in tissue at a body temperature of 37 °C, and a saprophytic, filamentous form at 25 °C) [8]. They live in plant material and soil of the environment. In addition, this fungus may result in zoonotic transmission and may infect some animals such as cats. The lesions appear after a trauma, often minor, inoculating the fungus. The main clinical forms are the cutaneo-lymphatic form and the fixed cutaneous form [8,9,10]. SPT is a cosmopolitan infection but is most commonly found in the tropical and subtropical areas of Brazil, India, Mexico, the USA, Japan, China, Madagascar, and South Africa [8,11,12,13,14,15,16,17,18,19,20,21]. The prevalence may reach 0.5% in some Latin American countries. The largest outbreak is currently occurring in Brazil, where more than 4000 cases were recorded in 2014. This zoonotic outbreak due to feline transmission is expanding to neighboring Brazilian regions [22]. In Europe, cases are rare. Only sporadic cases have been reported in France or Italy [23,24]. The standard diagnosis relies on histology and mycology. Although not specific, ‘asteroid bodies’ and the Splendore–Hoeppli reaction are highly suggestive of this diagnosis in histological analyses. Unfortunately, these elements are rare and, therefore, difficult to observe [8,9,25]. Mycological confirmation of the diagnosis is obtained by identifying *Sporothrix* spp. from cultures. Molecular diagnostics allow confirmation of the diagnosis and identification at the species level. The most common SPT pathogens belong to the *Sporothrix schenckii* sensu lato species complex, composed of the cryptic species *S. schenckii*, *S. brasiliensis*, *S. globosa*, *S. mexicana,* and *S. luriei* [8,11,12,16,17,21,26,27]. *S. schenckii* and *S. globosa* are cosmopolitan pathogens, whose transmission route is through wounds or plant stains [27,28]. *S. brasiliensis*, is associated with animal infections and zoonotic transmission through deep scratches and bites from infected animals [8,10,29,30]. *S. mexicana* and *S. luriei* are rarely described as the causative agent of SPT [27,31].

**Figure 1:**
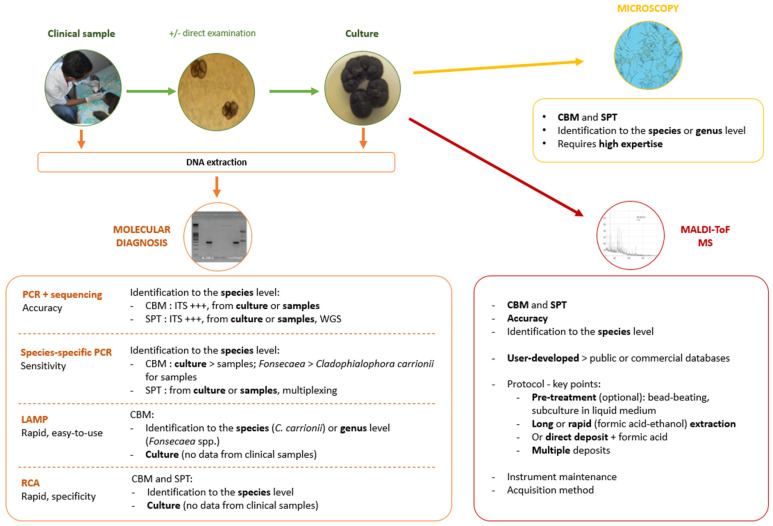
Diagnostic techniques for chromoblastomycosis (CBM) and sporotrichosis (SPT). ITS: internal spacer; WGS: whole genome sequencing; LAMP: loop-mediated isothermal amplification; RCA: rolling circle amplification; MALDI-ToF MS: matrix-assisted laser desorption/ionization time-of-flight mass spectrometry.

The laboratory diagnosis of these two implantation mycoses mainly relies on direct microscopy, histopathology, and isolation of the fungus by culture that can last days to weeks. While direct examination and culture are usually available in hospital laboratories of endemic countries, including local ones (because they are inexpensive), identification of the species by morphology requires mycological expertise that is lacking in peripheral health care facilities (Figure 1, Table 1). In addition, culture may result in false negatives, unreliable results due to environmental contamination, and delays in obtaining growth [1,5,8].

The molecular methods developed to detect and identify the causative agents can address the shortcomings in the morphological identification of the cultivated fungi, although not all can be made available in fieldwork or local laboratories, due to high costs or the need for specific equipment or expertise. In the present review, and based on our experience from Malagasy studies, we describe these methods based on PCR and sequencing. We also detail the use of the matrix-assisted laser desorption/ionization time-of-flight mass spectrometry (MALDI-TOF MS) and its application in identifying the fungal pathogens responsible for CBM and SPT [2,11]. We paid particular attention to the rapid and reliable methods that can be performed in remote laboratories and resource-limited conditions (Figure 1, Table 1).

Although the diagnostic issues regarding CBM and SPT are very close to those of eumycetoma, we decided not to deal with this third major implantation mycosis in this review, because of our more limited experience. In addition, molecular diagnosis of mycetoma has been recently and superbly reviewed in [32].

## 2. Chromoblastomycosis

### 2.1. Methods for Diagnosis and Identification from Culture Samples

#### 2.1.1. DNA Isolation

DNA extraction from fungal colonies can be performed either using in-house (including glass bead beating and chloroform–isoamyl alcohol) [33,34,35] or commercially available protocols, such as the Qiagen (QIAGEN, Venlo, The Netherlands) [2,36,37,38].

#### 2.1.2. DNA Sequencing

Various barcodes can be used in the rRNA genes (18S, 5.8S, and 28S rRNA), the two internal spacers (ITS) between them, or some protein-coding genes (translation elongation factor 1-α, β-tubulin, RNA polymerase II, calmodulin) [39,40]. The ITS1-5.8S-ITS2 region (referred after as the ITS region) is preferred to the rRNA genes, in order to identify a maximum of fungal species, and is recognized as the best candidate for a universal fungal barcode (Table 2). The protein coding genes may achieve better identification in certain fungal groups; however, their amplification and sequencing efficacies limit their use as a universal marker [40,41]. For CBM agents, ITS barcoding accurately differentiates the four species of the genus *Fonsecaea* implicated in CBM: *Fonsecaea pedrosoi*, *Fonsecaea monophora*, *Fonsecaea nubica*, and *Fonsecaea pugnacius* and differentiates them from *Cladophialophora carrionii* [2,36,37,38,42,43,44,45]. The nucleotide alignment of the ITS sequences enabled us to identify six *Cladophialophora carrionii* and 24 *Fonsecaea nubica* cases in our study ([2] and unpublished data).

Nevertheless, a multilocus analysis may be recommended for identifying the species and distinguishing between sibling species within the *Fonsecaea* genera by analyzing the ITS, the β-tubulin gene (BT2), the elongation factor 1α (TEF1 α), the Actin (ACT1), and Cell division cycle42 (Cdc42) genes [1,46,47,48,49] (Table 2).

**Table 2:** Primer sequences cited and used for sequencing or detection.

Target	Primers	Ref.
ITS	ITS1-CCGTAGGTGAACCTGCGG	[50]
	ITS4-TCCTCCGCTTATTGATATGC	[50]
	ITS3-GCATCGATGAAGAACGCAGC	[50]
	V9G-TTACGTCCCTGCCCTTTGTA	[51]
	LS266-GCATTCCCAAACAACTCGACTC	[51]
D1/D2	NL1-GCATATCAATAAGCGGAGGAAAAG NL4-GGTCCGTGTTTCAAGACGG	[52]
TEF1α	EF1-F CTGAGGCTCGTTACCAGGAG EF1-R CGACTTGATGACACCGACAG	[53]
ACT1	F-CACGTTGTCCCCATCTAC R-CACGTTGTCCCCATCTAC	[47]
BT2	BT2a-GGTAACCAAATCGGTGCTGCTT T2-TAGTGACCCTTGGCCCAGTTG	[54,55]
RPB1	F-GARTGYCCDGGDCAYTTYGG R-CCNGCDATNTCRTTRTCCATRTA	[40]
CAL	CL1-GA(GA)T(AT)CAAGGAGGCCTTCTC CL2A-TTTTTGCATCATGAGTTGGAC	[56]
CDC42	Cdc42-SF1s-GGCAAGACATGCTTGTTGATCTC Cdc42-SR1s-GCCTCGTCAAATACGTCCTTA A	[49]
*Cladophialophora* *Carrionnii*	CarF-TAAACCTCATGTTGCTTCG CarR-TCGAGAM(A/C)CACTCGACCAA	[57]
	CcarF-ATCGCTGCGAAGCGTCTCG Ccar-R 5′-ACCGTCCAACACCAAGCACAGG	[2]
*Fonseceae* spp.	Fon-F-TAATGCGGGTGTTGCCTCTG Fon-R-AGGGGTGGAAAGTGTGAACT	[58]
Lamp *Fonsecaea*	F outer (F3)-ACATTGCGCCCTTTGGTAT R outer (B3)-GCACCCTTCATCCGATACG F inner (FIP)-CAACACCAAGCACAGGGGCTTTTTCGAAGGGCATGCCTGTTC R inner (BIP)-TGGTGGAGCGAGTTCACACTTTTTTTAAAGAAGCTCAGTGTACCGG	[59]
Lamp *Cladophialophora*	F outer (F3)-CCGTCACGTGATTTCACACT R outer (B3)-CATCGATGACGGTGACGAAG F inner (FIP)-GAGCCCTTGCCGAGTTCAGCTTTCCCGAGCCTGATCAACT R inner (BIP)-ATGCCTGGGTTTTGGACAAGCTGTCTCGAACTTCCAGAGCG	[60]
RCA *Cladophialophora*	RCA1 ATGGGCACCGAAGAAGCA RCA2 CGCGCAGACACGATA padlock probe-specific-TCGGCGGACACGGGCCCAGAGatcaTGCTTCTTCGGTGCCCAT tacgaggtgcggatagctacCGCGCAGACACGATAgtctaAGAGTTTGGGGTTGGCTG	[60]
RCA *Fonsecaea*	Padlock probe-specific	[61]
RCA *Sporothrix*	padlock probe-specific	[15]

TEF1α: translation elongation factor 1-α; ACT1: actin; BT2: β-tubulin; Cdc42: cell division cycle42; RPB1: RNA polymerase II, CAL: calmodulin. F: forward; R: reverse; lamp RCA: rolling circle amplification.

#### 2.1.3. Nucleic Acid Amplification Tests (NAATs)

(a) Conventional NAATs (PCRs)

Species-specific PCRs have been developed to identify either *C. carrionii* [2,57] or species of the *Fonsecaea* genera (i.e., *F. pedrosoi* and *F. compacta*) [58] (Table 2). They showed specificity for species and genera, over various other relevant medically dematiaceous fungal species or pathogenic yeasts [57,58]. The *Fonsecaea* PCR targets a 333 bp-region within the ITS1. We confirmed that this PCR, initially developed by Abliz et al. to detect *F. pedrosoi* and *F. compacta* [58], can also detect *F. nubica*, even directly from clinical samples [2]. The *C. carrionii* PCRs target a 362 bp-fragment [57] or a 297 bp-fragment of the ITS region [2]. These specific PCRs showed sensitivity rates from culture of 92.3% (12/13) and 91.3% (35/39), respectively, for *C. carrionii* and *Fonsecaea* sp. detection ([2] and unpublished data).

(b) Rapid NAATs

(1) Loop-mediated isothermal amplification (LAMP)

LAMP is a DNA amplification method using a set of four or six specific primers that recognize six or eight distinct sequences of the target DNA [62]. Thus, it is extremely efficient and rapid. The isothermal conditions and the cycling reaction result in the accumulation of a very large amount of target copies in less than an hour. Furthermore, the products can easily be detected by the naked eye, due to the use of SYBR green that colors the positive wells in fluorescent green. The LAMP technology applied to the CBM diagnosis resulted in two methods designed to detect either the *Fonsecaea* genus or the *C. carrionii* species [59,60]. For *Fonsecaea* spp. the target was the ITS region, whereas for *C. carrionii* it was the *EF1* gene (Table 2). No cross-reactivity to related species or genera was observed but for *Fonsecaea*, LAMP could not distinguish between the different species of the genus, because they differ in relatively few nucleotide polymorphisms.

(2) Rolling circle amplification (RCA)

Similarly to LAMP, RCA-PCR is a simple isothermic amplification method. It is able to detect a single nucleotide polymorphism, which is not possible with a LAMP assay. Thus, RCA is more specific than LAMP, but LAMP is more sensitive than RCA. An RCA method using three specific padlock probes targeting the ITS region was shown to specifically identify the three sibling *Fonsecaea* species involved in CBM (i.e., *F. pedrosoi*, *F. nubica*, and *F. monophora*) [61]. Similarly, an RCA reaction, also targeting the ITS region, was designed to specifically identify C. *carrionii* [60] (Table 2).

#### 2.1.4. MALDI-ToF Mass Spectrometry

Comparing the highly specific protein spectra generated by MALDI-ToF MS with formerly created databases (‘libraries’) makes it possible to identify cultured microorganisms up to the species level, within a few min. The accuracy of this identification relies, both on the quality of the generated spectra (itself dependent on culture and extraction methods), and on the number of reference spectra included in the library.

The use of MALDI-TOF MS in the context of CBM diagnosis requires the growth of a pure culture from a skin sample, which in the case of CBM, can be easily contaminated by environmental molds. Once the culture has grown, different protein extraction methods can be used according to the manufacturer’s instructions. Indeed, the quality of the spectra and the rate of correct identification are usually improved by using long extraction protocols, which were also used for the creation of the databases. For identification with the MALDI Biotyper^®^ (Bruker Daltonics GmbH, Bremen, Germany), Bruker recommends direct extraction from the solid medium in the first instance, but if this rapid procedure fails, an off-plate extraction protocol (also called ethanol-acid formic extraction), requiring an additional ten min, is proposed from the same solid medium. As a last resort, culture in liquid medium can be carried out, but this protocol extends the identification time by 24 h. When using the National Institutes of Health database, bead beating of the culture followed by the ethanol-acid formic extraction is recommended [63]. In our experience, both rapid acid-formic-ethanol extraction and direct deposit with formic acid have yielded accurate identification results with our homemade library [64]. Multiple deposits (minimum 3 or 4) on the ground-steel targets is also very helpful.

The scarcity, or the absence of, reference spectra in commercial libraries complicates identification of CBM agents, even in the presence of high quality spectra. Regular updates of these databases by manufacturers should fill this gap over time, but in the meantime, creating homemade libraries, even if time-consuming, remains the best solution for implementing rare fungal species. We used this strategy to rapidly identify CBM causative agents in Madagascar [2] and generated 11 reference spectra with a Microflex Mass Spectrometer (Bruker Daltonics GmbH, Bremen, Germany) from reference or previously sequenced strains of *C. carrionii*, *F. nubica*, *F. pedrosoi*, and *F. monophora* [2]. These homemade spectra allow us to, now, precisely and quickly identify these agents on cultures, without the need for DNA sequencing. In their study on melanized fungi identification, including some CBM agents, Paul S. et al. also concluded that the use of in-house libraries allows faster and more accurate identification, with 100% (117/117 cultures) correct identification vs. 29/117 (24.8%) with the Bruker library [65].

Another clear limitation of MALDI-TOF MS in the context of CBM diagnostics is the obvious impossibility of identifying the implicated pathogen directly on the clinical sample. Indeed, there is currently no efficient extraction protocol applied directly to complex clinical samples, such as human skin. Moreover, fumagoïd cells may be present in the skin sample but their number is limited, challenging the fungal protein specific extraction. Lastly, there is simply no library built from fumagoïd cells, because this state is only found in vivo.

Lastly, instrument performance variability can also be a limitation for accurate identification of rare filamentous fungi. In their 2019 multicenter study, Lau et al. also suggested adapting the acquisition method of the instrument and proposed a mold-specific acquisition method that improves results and reproducibility across instruments. Correct and regular maintenance of the instrument is also mandatory [66].

### 2.2. Methods for Diagnosis from Clinical Samples

These methods are very beneficial, as they anticipate or bypass the culture step and may save time-to-results, in addition to being more sensitive and specific.

#### 2.2.1. Skin or Subcutaneous Samples (Squamous, Biopsies, Needle Aspirates)

(a) DNA isolation

For CBM diagnosis, clinical samples can be cut into small pieces, mixed with glass beads, and then crushed by intense vortexing. DNA can then be purified using commercial assays, such as the QIAamp DNA Blood Mini Kit (QIAGEN, Venlo, The Netherlands) [2].

(b) DNA sequencing

It is possible to detect and then identify the fungal agents responsible for CBM directly from clinical specimens, by amplifying and then sequencing universal DNA barcode markers. The markers described above, which were developed from cultivated strains, can be used from clinical samples.

For CBM diagnosis, we compared the ITS region to the D1/D2 one (part of the 28S rRNA). From 50 clinical samples, we found that ITS amplification (using the ITS1-ITS4 universal primers) was less sensitive than D1/D2 (using the NL1-NL4 universal primers, Table 2). However, the ITS sequences subjected to the International Society of Human and Animal Mycology (ISHAM) database resulted in a more accurate identification of the *Fonsecaea* spp (there was no difference for *Cladophialophora carrionii* identification). Indeed, the D1/D2 sequences were not suitable for correct identification, mainly because the National Center for Biotechnology Information (NCBI) database requires an upgrade [2].

(c) Nucleic acid amplification tests (NAATs)

(1) Conventional NAATs (PCRs)

The species-specific PCRs specific to the *Fonsecaea* genus and to *C. carrionii* described above were initially developed to detect *F. pedrosoi*, *F. compacta,* and *C. carrionii* from cultivated strains. Both *C. carrionii* and *Fonsecaea* spp. PCRs detected the fungi directly from clinical samples [2]. As regards the sensitivity of these PCRs for the clinical specimens that we collected in Madagascar, we observed that the PCR for *C. carrionii* was unable to detect the fungus from any of the specimens collected from the 13 cases of CBM due to this species. In contrast, the PCR for *Fonsecaea* spp. established a diagnosis in 21 (55.2%) clinical samples for the 38 cases caused by this genus ([2] and unpublished data).

(2) Rapid NAATs

LAMP and RCA were developed from cultivated strains and have not yet been applied directly with clinical samples. As RCA and LAMP are sensitive, specific, and enable reproducible isothermal DNA amplification, their use from clinical samples after DNA extraction would represent a significant improvement in CBM diagnosis. Thus, further studies are needed to evaluate their performances and their feasibility in the field.

#### 2.2.2. Formalin, Fixed and Paraffin Embedded (FFPE) Tissues

(a) DNA isolation

A study that compared five commercial methods for DNA extraction established that, from 25-µm thick sections of a paraffin block, the best assay was the TaKaRa Dexpat Kit (Takara Bio Inc., Shiga, Japan), which achieved a sensitivity of 91% in purifying DNA for fungal detection [67].

(b) DNA sequencing

Sequence-based protocols for detecting fungi in FFPE tissue, target either the 18S rRNA gene, the 28S rRNA gene, or ITS fragments. In a study that compared DNA extraction protocols, three pan-fungal PCRs were evaluated and found to achieve the best efficiency when amplifying the ITS2 region using the ITS3 and ITS4 primers [67]. However, they have mainly been evaluated for invasive fungal infections, and rarely for implantation ones. One study analyzed 17 PFFE samples of tropical fungal infections, including three with histopathology evidence of CBM [68]. Using the best extraction protocol cited above and described in [67], the authors used five pan-fugal PCRs targeting 18S rRNA gene, the 28S rRNA gene, or ITS, including the one selected in [67]. None of these protocols successfully identified the CBM fungus. Indeed, as with the other diagnosis in this study, the number of environmental fungi was high and the authors thought that they had probably been deposited on the paraffin blocks and contaminated the samples.

## 3. Sporotrichosis

### 3.1. Methods for Diagnosis and Identification from Culture Samples

#### 3.1.1. DNA Isolation

This technique uses mechanical grinding from culture, followed by conventional extraction using commercial extraction kits, such as the Qiagen (QIAGEN, Venlo, The Netherlands) [2,11].

#### 3.1.2. DNA Sequencing

The use of a gene or group of genes is recommended for distinguishing species belonging to the *S schenckii* complex, such as *S. brasiliensis*, *S. mexicana*, *S.globosa*, *S. schenckii*, *S. luriei*, and *S. albicans.* These include the CAL, BT2, and chitin synthase (CHS) genes, and the ITS or 28S large subunit (LSU) [11,69,70] (Table 3). In addition, confirmation at the species level can be achieved by observing the clustering of sequences by clade, in relation to reference sequences in a phylogenetic tree. This has been achieved by targeting the ITS or CAL gene [11,70].

In our Malagasy study, D1/D2 and ITS sequences were used for species identification. The D1/D2 domain did not allow identification at the species level, whereas ITS sequencing identified 34 *S. schenckii* strains ([11] and unpublished data).

Multi-gene analyses based on ITS, 5.8s region, BT2, CAL, and TEF1α increase the accuracy of the taxonomic resolution of species within the clade. They are particularly useful for recognizing rare agents, such as *S. chilensis* (a member of the *S. pallida* complex) and *S. mexicana* (a rare soil-borne species, not pathogenic to humans [71] (Table 3). Among these target sequences, the ITS region is the primary barcode marker for the diagnosis of species in the clinical group [16,71].

Sequencing the whole genome allows the detection of possible variants of the sporotrichosis causative agents [72].

#### 3.1.3. Nucleic Acid Amplification Tests (NAATs)

(a) Conventional NAATs (PCRs)

The region covering exons 3 to 5 of the CAL gene is the main marker used for the recognition of *Sporothrix* spp. Species-specific primers that selectively amplify *Sporothrix* species are available with an amplicon size of 469 pb, 331 pb, 243 pb, 183 pb, and 363 pb, respectively, for *S. brasiliensis, S. schenckii, S. globosa, S. mexicana,* and *S. pallida* [73] (Table 3 and Table 4).

In our study, we targeted the topoisomerase II gene with the SSHF31-SSHR97 primer pair, amplifying a size of 663–817 bp for the detection of *Sporothrix* spp. [11,75] (Table 4). We obtained a sensitivity rate for the *Sporothrix* spp. detection of 91.4% (74/81) from the culture of the SPT cases ([11] and unpublished data).

Recent studies have used the multiplex PCR technique, targeting the CAL gene for simultaneous species detection: *S. globosa*, *S. schenckii*, and *S. brasiliensis*. The sensitivity and specificity reached 100% [13]. A multiplex qPCR assay amplifying the BT2 gene has also been developed and evaluated for the rapid diagnosis of emerging sporotrichosis caused by *S. brasiliensis*, *S. schenckii,* and *S. globosa* [14] (Table 3).

(b) PCR-RFLP, PCR-RAPD

Identifying *Sporothrix* by PCR-RFLP targeting the CAL gene digested with the HhaI endonuclease shows five specific restriction profiles to distinguish the main species: *S. brasiliensis*, *S. schenckii*, *S. globosa*, *S. mexicana*, *S. pallida,* and *S. luriei* [73] (Table 3).

(c) Rapid NAATs: rolling circle amplification (RCA)

This method is based on lock probe amplification; i.e., linear DNA probes become circular (via the enzyme Pfu DNA ligase) when a given sequence is specifically recognized. RCA can be applied in pure culture but also from complex environmental samples, such as soil and plants [15] (Table 2 and Table 3).

#### 3.1.4. MALDI-ToF MS

As detailed for CBM pathogen identification with MALDI-ToF MS (cf supra), the use of this powerful technology can theoretically identify any *Sporothrix* sp. responsible for sporotrichosis. The same prerequisites as for CBM agents are needed to correctly identify the *Sporothrix* species (i.e., correct and regular maintenance of the instrument, adapted protein extraction, use of multiple deposits (3 or 4), optimized spectra acquisition methods, presence of reference spectra in the commercial or homemade library). In 2015, Oliveira et al. developed a new MALDI-TOF protocol for identifying clinical and environmental isolates of the six species belonging to the *Sporothrix* complex on Axima LNR equipment (Kratos Analytical, Shimadzu), with the SARAMIS^®^ software package (Spectral Archiving and Microbial Identification System; AnagnosTec, Postdam-Golm, Germany) [76]. This enriched library allowed accurate identification of 64/64 (100%) clinical and environmental isolates. Subsequently, Matos et al. successfully used the same enriched database for diagnosing ocular sporotrichosis [75]. In the multicenter study from Lau et al., comparing the efficacy of fungal identification between eight centers, among 80 clinical isolates, two strains of *Sporothrix schenckii* were sent to the different centers and tested with both Bruker (V3.0) and NIH databases [66]. As the NIH database failed to identify these strains, the Bruker database misidentified *S. schenckii* as *Candida chodatii,* highlighting the need to enrich both databases with *Sporothrix* species reference spectra. In 2013, we began a large epidemiological study on sporotrichosis in Madagascar [11]. At that time, reference spectra were scarce in the available databases (2 MSPs in the Bruker database, and 1 profile in the NIH database for *Sporothrix* sp.). We, thus, generated 20 main spectra profiles (MSP) from reference or previously sequenced strains of *Sporothrix schenkii* [11]. This homemade library was successfully used for identifying *S. schenkii* in our study [11]. In 2018, as an external control, all our identifications and reference spectra were compared with the freely available and external user-developed online platform Mass Spectrometry Identification (MSI, available online at https://msi.happy-dev.fr/ (accessed on 1 April 2022)), which confirmed 100% of our *S. schenkii* identifications [11]. Of note, the open-access MSI database is, as far as we know, the only database which freely provides a significant number of reference spectra for the identification of *Sporothrix schenkii* and its related cryptic species (12 reference spectra for *S. schenkii,* and 4 for *S. braziliensis* and *S. globosa*) [77]. The Microbenet database, developed by the CDC (https://microbenet.cdc.gov; accessed on 1 April 2022) is also freely available online for MALDI-ToF MS identification of rare microorganisms, but we were unable to find information on its performance in the context of rare filamentous fungi diagnosis.

### 3.2. Methods for Diagnosis from Clinical Samples

Skin or Subcutaneous Samples (Squamous, Biopsies, Needle Aspirates, …)

(a) DNA isolation

The same method as described above for CBM can be used [2,11].

(b) DNA sequencing

Sequencing a region amplified by universal primers allows the detection of fungal agents responsible for SPT. As for CBM, we compared the ITS region with the D1/D2 one. We found that amplifying the D1/D2 region was more successful in terms of the sensitivity and the quality of the sequences we obtained; however, as for CBM species, the ITS sequencing resulted in more accurate identification of the *Sporothrix* species [11].

(c) Nucleic acid amplification tests (NAATs)

(1) Conventional PCRs

We used PCR of the topoisomerase II gene with the specific primer pair of *S. schenckii* and obtained a sensitivity of 3.1% from the 65 clinical samples tested [11]. This suggests that the topoisomerase II gene may not be a good target for detecting *S. schenckii* directly in clinical samples.

Multiplex qPCR assay directly from DNA extracted in tissue biopsy is available, for rapidly detecting *S. globosa*, *S. schenckii*, and *S. brasiliensis*. The target gene is the CAL gene, with a positive detection rate of 93.9% [13]. The ITS region is also used in a real-time PCR method for *S. globosa* from tissue samples, for fast clinical diagnosis [78].

(2) Rapid NAATs

RCA was developed from cultivated strains and has not yet been applied directly to clinical samples. As RCA is a sensitive, specific, and reproducible isothermal DNA amplification, its use from clinical samples after DNA extraction would represent a significant improvement in SPT diagnosis. Thus, further studies are needed to evaluate its performances and feasibility in the field.

All the above tools are described for the diagnosis of the main subcutaneous clinical forms of SPT (i.e., fixed cutaneous and lymphocutaneous) and can be used with skin or subcutaneous samples. For disseminated or extracutaneous forms, the same molecular methods, either from cultures or directly from samples, may be applied. Clinical samples may be mucous membrane samples (for ocular forms or mucosal lesions in disseminated forms), respiratory samples (for lung involvement, due to inhalation or hematogenous route), and bone and joint samples (for the osteoarticular form due to traumatic or hematogenous route).

## 4. Conclusions and Perspectives

Molecular methods for diagnosing CBM and SPT may increase the sensitivity of the detection of the fungal agents from clinical specimens and culture. In addition, they improve the accuracy of fungal identification at the species level, resulting in the discrimination of sibling species of the *Fonsecaea* or the *Sporothrix* genera that cannot be distinguished by morphological features. Indeed, correct identification has both epidemiological and clinical impacts. As an example, DNA sequencing led to a revision of *F. nubica,* previously described as *F. pedrosoi,* as the main agent responsible for CBM in Madagascar [2]. This led to the Maldi-ToF databases being expanded, in order to further facilitate and improve diagnosis; and, thus, patient management. For SPT, the rapid identification of the emerging zoonotic and highly pathogenic species is required for optimal treatment. Progresses in diagnosing CBM and SPT using these molecular techniques will help in the evaluation of their actual burden, and may have a major impact on public health policies, to improve patient management.

Now, these techniques must make the leap from the research or reference laboratories, to the peripheral facilities in regional and district structures, in order to reach the rural and remote populations who are targeted. The main limitation of the molecular diagnosis of CBM and SPT is its cost, which includes the need for specific equipment and the presence of trained laboratory employees to perform these techniques efficiently. This cost is still prohibitive for the health systems in endemic countries. Although simple and accurate, MALDI-TOF MS is not well adapted to these settings: it still requires culture, reference spectra are lacking in the common databases, and the equipment is rather expensive. Recent technologies based on isothermal amplification and fluorescent detection visible to the naked eye offer easy-to-use, rapid, and cost-effective alternatives to the usual PCR techniques. Multiplex systems may resolve the challenge of multiple species detection. In addition, these techniques need to be applied directly from clinical samples, to avoid delays and the possible contamination of the culture performed in poorly equipped laboratories. Another challenging and limiting step will be implementing these tools in peripheral facilities (Table 1). This can only be achieved if (i) the laboratory technicians and biologists are sufficient in number and fully trained to use these new tools, (ii) the supply of these laboratories is guaranteed, and (iii) there are national programs that are well funded and structured to support these actions. The recognition of these two fungal infections as NTDs by the World Health Organization is an encouraging signal to help endemic countries initiate such programs.

## Figures and Tables

**Table 1:** Availability of molecular diagnostic techniques of chromoblastomycosis and sporotrichosis.

		Fieldwork/Rural Clinics	Local Laboratories	Expert Laboratories
Conventional Diagnosis	Microscopy	X	X	X
	Culture		X	X
Molecular Diagnosis	DNA Amplification	To be achieved	X	X
		From clinical samples	From culture or clinical samples	From culture or clinical samples
		Easy-to-use methods (ex: LAMP)	Easy-to-use methods (ex: LAMP)	
	DNA Sequencing			X
	MALDI-ToF MS			X
Development of New Diagnostic Tools				X

LAMP: loop-mediated isothermal amplification MALDI-ToF MS: matrix-assisted laser desorption/ionization time-of-flight mass spectrometry.

**Table 3:** Molecular techniques developed to diagnose sporotrichosis.

Technique	Target	Ref.
Gene sequencing	CAL, BT2, CHS, ITS, 28S large subunit (LSU),	[11,69,70]
Multi-gene sequencing (i.e., MLST)	ITS, 5.8S, BT2, CAL, TEF1α	[16,71]
Conventional PCR	CAL, topoisomerase II	[73,74]
Multiplex PCR	CAL, BT2	[13,14]
PCR-RFLP	CAL	[73]
RCA	CAL	[15]

**Table 4:** Primer sequences and amplicon sizes for the detection of *Sporothrix* species and *Sporothrix* spp by conventional PCR.

Primers	Detection	Amplicon Size	Target Gene	Ref.
Sbra-F-CCCCCGTTTGACGCTTGG	*S. brasiliensis*	469 pb	CAL	[73]
Sbra-R-CCCGGATAACCGTGTGTC ATAAT				
Ssch-F-TTTCGAATGCGTTCGGCTGG	*S. schenckii*	331 pb	CAL gene	[73]
Ssch-R-CTCCAGATCACCGTGTCA				
Sglo-F-CGCCTAGGCCAGATCACCACTAAG	*S. globosa*	243 pb	CAL gene	[73]
Sglo-R-CCA ATG TCT ACC CGT GCT				
Smex-F-TCTCTGCCGACAATTCTTTCTC	*S. mexicana*	183 pb	CAL gene	[73]
Smex-R-GGAAAGCGGTGGCTAGATGC				
Spa-F-CGCTGCTTTCCGCCATTTTCGC	*S. pallida*	363 pb	CAL gene	[73]
Spa-R-GCCATTGTTGTCGCGGTCGAAG				
SSHF31-GCAGCCCACGTCCAACAAGACT	*Sporothrix* spp.	663−817 bp	topoisomerase II	[11,74]
SSHR97-GTCAGAGGTCTTATTGGACGTGA				

## Data Availability

Not applicable.
